# Involvement of Host Non-Coding RNAs in the Pathogenesis of the Influenza Virus

**DOI:** 10.3390/ijms18010039

**Published:** 2016-12-27

**Authors:** Yanmei Ma, Jing Ouyang, Jingyun Wei, Mohamed Maarouf, Ji-Long Chen

**Affiliations:** 1 College of Animal Sciences, Fujian Agriculture and Forestry University, Fuzhou 350002, China; 89mym@163.com (Y.M.); Jessieweijy@hotmail.com (J.W.); 2 CAS Key Laboratory of Pathogenic Microbiology and Immunology, Institute of Microbiology, Chinese Academy of Sciences (CAS), Beijing 100101, China; wsmagg@hotmail.com (J.O.); mohamed_maarof@im.ac.cn (M.M.); 3 International College, University of Chinese Academy of Sciences, Beijing 100101, China

**Keywords:** non-coding RNAs, influenza A virus, microRNAs, lncRNAs, vault RNAs

## Abstract

Non-coding RNAs (ncRNAs) are a new type of regulators that play important roles in various cellular processes, including cell growth, differentiation, survival, and apoptosis. ncRNAs, including small non-coding RNAs (e.g., microRNAs, small interfering RNAs) and long non-coding RNAs (lncRNAs), are pervasively transcribed in human and mammalian cells. Recently, it has been recognized that these ncRNAs are critically implicated in the virus–host interaction as key regulators of transcription or post-transcription during viral infection. Influenza A virus (IAV) is still a major threat to human health. Hundreds of ncRNAs are differentially expressed in response to infection with IAV, such as infection by pandemic H1N1 and highly pathogenic avian strains. There is increasing evidence demonstrating functional involvement of these regulatory microRNAs, vault RNAs (vtRNAs) and lncRNAs in pathogenesis of influenza virus, including a variety of host immune responses. For example, it has been shown that ncRNAs regulate activation of pattern recognition receptor (PRR)-associated signaling and transcription factors (nuclear factor κ-light-chain-enhancer of activated B cells, NF-κB), as well as production of interferons (IFNs) and cytokines, and expression of critical IFN-stimulated genes (ISGs). The vital functions of IAV-regulated ncRNAs either to against defend viral invasion or to promote progeny viron production are summarized in this review. In addition, we also highlight the potentials of ncRNAs as therapeutic targets and diagnostic biomarkers.

## 1. Introduction

One of the most important discoveries contributed by transcriptome projects ENCODE and GENCODE is the finding that more than 85% of human genome are transcribed to RNA, but less than 3% of genome encode proteins, and thus most transcripts of human genome are non-coding RNAs (ncRNAs) without protein-coding capacity [1,2]. In the most recent GENCODE release, there were 7258 small non-coding RNA genes in human genome, encoding microRNA (miRNA, ~22 nt), small interfering RNA (siRNA, 21–25 nt), piwi-related RNA (piRNA, 24–33 nt), vault RNAs (vtRNAs, 80–150 nt) and other small RNA molecules (100–200 nt) [3,4,5]. The number of human long non-coding RNA genes (lncRNAs, >200 nt) is 15,767, in addition to 14,650 pseudogenes. These non-coding portions of the genome were found to be highly related to the evolution complexity of organisms [6,7]. In the last 20 years, there has been growing data showing that not only the proteins, but also many non-coding RNAs act as key regulators of multiple cellular processes.

The influenza virus is a negative single-strand RNA virus belonging to the Orthomyxoviridae family [8]. Based on the different antigenicities of nucleic protein (NP) and matrix protein (M), the influenza virus is mainly divided into three serotypes (A, B and C). Influenza A virus (IAV) is the dominant pathogen of influenza that caused four pandemics as well as causing annual seasonal epidemics, leading to enormous morbidity and economic loss annually in the world [9]. IAVs are classified to 16 hemagglutinin (HA) subtypes (H1 to H16) and 9 neuraminidase (NA) subtypes (N1 to N9) [10]. All of these subtypes are observed in aquatic birds [8]. The unusual new H17N10 and H18N11 identified in bats are completely different to the circulating IAV strains [11]. Remarkably, H5N1 and H7N9 are two subtypes that can infect human and cause highly pathogenic influenza. H1N1 is an important subtype that caused two pandemics, in 1918 and 2009, threatening the health of over millions of people. After infecting the host, the influenza virus damages the respiratory epithelium, lung epithelial cells, with symptoms including headache, fever, cough, pneumonia, and seriously even death [10].

Upon IAV infection, host cells can detect the viral pathogen-associated molecular pattern (PAMPs) through pattern recognition receptors (PRRs), such as toll-like receptor 3 (TLR3), retinoic acid-inducible gene 1 (*RIG-I*), and melanoma differentiation-associated gene 5 (*MDA5*) [12,13]. Subsequently, series cascades of transcription and activation in innate immune response are triggered, including the PRR-dependent signaling pathways, production of IFNs and other cytokines, and expression of antiviral ISGs [14]. These proteins not only inhibit virus replication in the infected cells, but also recruit dendritic cells and macrophages to virus infected tissues, and further stimulate the immune response mediated by T cells and B cells [15].

Over the past ten years, it has been established that ncRNAs act as an important class of regulators involved in virus–host interaction, especially in the antiviral immune response [16]. In this review, we summarize the roles of ncRNAs, including microRNAs, lncRNAs and vtRNAs, in the influenza virus pathogenesis. Moreover, the mechanisms underlying involvement of these ncRNAs in the virus infection will also be discussed.

## 2. Biology of ncRNAs

### 2.1. miRNAs

Over 1000 miRNAs are encoded in the human genome, and target more than half of the human protein-coding genes [17]. Most miRNA genes are transcribed by RNA polymerase II and form long primary miRNAs (pri-miRNAs) with a 5′-cap, 3′-polyadenylated tail and several ~80 nucleotide (nt) hairpins [18,19,20,21,22,23]. After the cleavage by RNase III enzyme Drosha or other enzymes, precursor miRNAs (pre-miRNAs) of ~60 nt with a stem-loop structure are produced in the nucleus, and then transported to cytoplasm by exportin 5 [24]. The pre-miRNAs and many associated proteins form an active RNA-induced silencing complex (RISC) containing Dicer [19,23,25,26]. With the digestion by enzyme Dicer, the mature ~22-nt miRNAs are produced. Most miRNAs function through the canonical mechanism based on annealing to the 3′-untranslated region (3′-UTR) of the target mRNAs, and silence the gene post-transcriptionally by inducing mRNA degradation or inhibiting the translation. In some exceptions, miRNAs bind to coding region to regulate expression of the target genes [25].

### 2.2. lncRNAs

lncRNAs are newly recognized important regulators. They are products of RNA polymerase II or III, longer than 200 nt, with 5′-cap, and with or without 3′-polyadenylated tails. Based on the difference of the genomic locations and their relationship with neighboring protein-coding genes, lncRNAs are approximately classified as sense, antisense, bidirectional, intronic, and intergenic lncRNAs [27]. lncRNAs can fold to specific three-dimensional structures that interact with many kinds of large biological molecules, including genomic DNA, microRNA, mRNA and proteins, and are distributed in nucleus, cytoplasm, or even in exosome [28]. Recently, more and more lncRNAs have been identified as novel important regulators implicated in a wide range of cellular processes through various mechanisms by which they function as signaling, decoying, guiding, and scaffolding [13].

### 2.3. vtRNAs

vtRNAs are small non-coding RNAs with a length from 80 to 150 nucleotides produced by RNA polymerase III. They are a portion of cytoplasmic particle vaults, the large ribonucleoprotein particles with unusual barrel shaped morphology [29,30]. In a cryo-electron microscopy study, vtRNAs were seen in a structure close to the end caps of vaults [31]. However, most of vtRNAs are in a free form in cytoplasm [31,32]. Human vtRNAs are composed of vtRNA1-1, vtRNA1-2, vtRNA1-3, and vtRNA2-1 (also known as nc886). They are abnormally highly expressed in cancer cells and associated with drug resistance. vtRNAs are shown to be processed by Dicer to form small vault non-coding RNAs. It has been revealed that these small RNAs function in silencing cytochrome P-450 monooxygenase 3A4 (CYP3A4), a key enzyme in drug metabolism [33]. Recently, vtRNAs are found to be critically involved in regulating protein kinase RNA (PKR) activation, and thus they act as a novel suppressor of tumors and appear to play a key role in inhibition of innate immune response to IAV infection [34,35,36]. The expression of vtRNAs are significantly upregulated after viral infection, including IAV and γ-herpesviruses epstein barr virus (EBV) [32,35,37], indicating the importance of vtRNAs in the interaction between host and virus. However, to date, we still know little about the precise mechanism underlying regulation of vtRNA expression during viral infection.

## 3. Critical Roles of miRNAs in Influenza A Virus (IAV) Infection

### 3.1. Functional Involvement of miRNAs in Host–Virus Interaction

It is well known that small non-coding miRNAs play important roles in various biological processes. Recently, numerous data have revealed that miRNA networks profoundly influence the immune homeostasis or pathological immune response through post-transcriptional mechanisms [38,39,40,41]. Moreover, many host miRNAs have been reported to act as important regulators involved in antiviral response [42,43,44], leading to inhibition of viral replication through targeting host or virus genes. For example, microRNA-32 (miR-32) serves as an antiviral molecule against primate foamy virus (PFV) in infected Hela cells [45]. Mice with lower Dicer activity are more susceptible to vesicular stomatitis virus (VSV) due to impairment of miR24 and miR93 expression [46]. miR-198 was found to inhibit the replication of human immunodeficiency virus type 1 (HIV-1) by suppressing Cyclin T1 protein (CCNT1) expression [47]. In human T-cell lymphotropic virus type 1 (HTLV-1)-infected cells, many of the cellular miRNAs, such as miR-223, miR-181a, miR-155, and miR-125a, are modulated to enhance host innate immunity or promote T-cell differentiation [48]. In addition, some miRNAs directly target viral gene expression. For example, miR-125b and miR-223 directly inhibit the mRNA of human immunodeficiency virus type 1, resulting in a decrease of viral gene expression in resting cluster of differentiation 4 (CD4) T cells [43]. On the other hand, however, some host miRNAs could be hijacked by virus to facilitate its infection. For example, miR-122 is critical for RNA replication and translation initiation of hepatitis C virus (HCV) [49,50]. miR-100 and miR-101 are also been manipulated by human cytomegalovirus (HCMV) to enhance the viral replication [51]. Interestingly, it has been shown that some viral miRNAs could also be produced to establish a successful infection, such as expression of adenoviral miRNAs and herpes simplex virus (HSV)-1 miRNA, miR-H1 [52,53].

### 3.2. miRNAs Play Important Roles in Antiviral Response to IAV Infection

**Figure 1:**
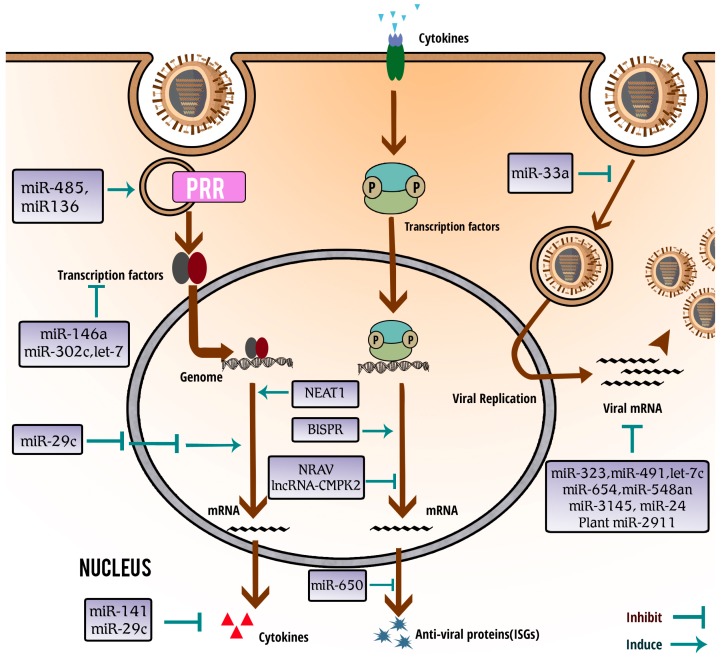
Host and viral ncRNAs regulate host innate immune response and viral infection. IAV invades the cells and replicates itself using the cellular machine. After sensing the invading virus via PRRs, host cells trigger the activation and nuclear translocation of transcription factors and produce robust IFNs and various cytokines. In turn, the IFNs and cytokines initiate the receptor-associated signaling pathways that lead to the production of antiviral proteins (e.g., ISGs) to defend against viral invasion. Many functional miRNAs and lncRNAs, including nuclear enriched abundant transcript 1 (NEAT1), bone marrow stromal antigen 2 (BST2) IFN-stimulated positive regulator (BISPR), negative regulator of antiviral response (NRAV) and lncRNA cytidine/uridine monophosphate kinase 2 (lncRNA-CMPK2), are evidenced to be involved in these signaling pathways by regulating different steps in order to inhibit or promote the IAV replication.

Previous studies have profiled the expression of miRNAs during IAV infection and found that abundance of more than one hundred miRNAs were significantly changed in infected cells [54,55,56,57]. Compared with the total number of miRNAs in human cells (predicted to be around 1000), it is surprising that approximately 10% of miRNAs are differentially expressed after IAV infection [56,58,59]. Among these miRNAs, dozens have been identified to play important roles in host innate antiviral response to IAV infection. They appeared to inhibit the viral infection (e.g., miR-4276 [60] and miR-650 [61]) or to facilitate (e.g., miR-451 [62] and miR-548an [63]) IAV replication (Figure 1 and Table 1). Herein, we summarize the critical miRNAs involved in IAV-host interaction according to their regulatory functions in innate immune response, and discuss the potential applications of these miRNAs as novel diagnosis and therapy tools, or novel biomarkers for severe influenza.

### 3.3. Influenza Virus Infection Induces Differential Expression of Distinct miRNAs

Differential expression profiles of host miRNAs in response to infection with various IAV strains (avian, murine, or human) have been revealed through in vivo and in vitro studies using microarray or next generation sequencing. Furthermore, co-expression and Gene Ontology (GO) analysis of target genes of miRNAs has established their important roles in regulating innate immune signaling, inflammation, cell differentiation, cell cycle and apoptosis during the IAV infection [26,64,84,85,86,87,88].

For instance, a distinct profile with 130 murine miRNAs significantly changed in response to infection with reconstructed 1918 influenza virus (r1918) has been reported through a microRNAome study. Intriguingly, 18 of these miRNAs were indicated to target host genes that crucially contribute to the extreme lethality of r1918 virus, resulting in increased IFN response or altering cell survival [85]. Compared with seasonal IAV strain A/Texas/36/91 (H1N1), r1918 causes strong downregulation of miR-193, miR-29a and miR-29b, while Texas/91 causes massive up-regulation of miR-193, and modest downregulation of both miR-29a and miR-29b. Another biomarker, miR-709, is upregulated by r1918 but strongly downregulated by Texas/91 infection [85]. Recently, in human patients of the highly pathogenic H7N9 avian influenza, 153 human serum miRNAs (146 enhanced and 7 suppressed) were detected by miRNA array and their potential as biomarkers for diagnosis were suggested [89]. In addition, it was identified that in lung tissues from infected cynomolgus macaques, 23 miRNAs were associated with the extreme virulence of the highly pathogenic H5N1 avian influenza [87].

### 3.4. miRNAs Regulate Interaction between Host and Influenza Virus

Various miRNAs are involved in host–IAV interaction through targeting host immune genes and modulating the host immune response signaling pathways, immune cell differentiation, cell cycle and so on (Figure 1 and Table 1). Host senses IAV infection by PRRs, including RIG-I, MDA5 and TLR3. The dual functional miR-485 was found to directly target RIG-I mRNA, resulting in degradation of the mRNA. In cells infected with a low abundance of H5N1 virus, miR-485 suppresses the antiviral response and enhances viral replication [75]. It was also observed that RIG-I activity was downregulated by enterovirus 71 though virus-mediated decrease of miR-526a and increased expression of its target cylindromatosis (CYLD), a negative regulator of RIG-I activity [90]. Another miRNA, H5N1-induced host miR-136, was indicated as an immune agonist of RIG-I, causing IL-6 and IFN-β accumulation in A549 cells. However, other reports showed that miR-136 inhibited IL-6 expression by binding with 3′-UTR of IL-6 [71], and the mechanism underlying action of miR-136 remains to be clarified. Moreover, many important components of innate immune system, including IRAK1 and MAPK3, are thought to be targeted by IAV-induced miRNAs (e.g., miR-7, miR-132, miR-146a, miR-187 and miR-1275) [64]. In consideration of the crucial roles of IRAK1 and MAPK3, it would be worthwhile to further define the function of these miRNAs in an innate immune signal transduction system.

NF-κB is one of the most important transcription factors implicated in the innate immunity. Several miRNAs were reported to be associated with the activation or translocation of NF-κB. For example, the known NF-κB activator, TRAF6, as a target of miR-146a, is downregulated in NK or T cells by overexpressed miR-146a [91,92]. Previous investigation found that miR-146a was upregulated by both H1N1 and H3N2, in addition to the infection of VSV, EBV and HTLV [64,73]. However, inhibition of miR-146a unexpectedly resulted in significant increase of both H1N1 and H3N2 propagation. Therefore, virus-induced miR-146a might have function in the crossroad of immune response, and not only suppresses TRAF6 expression and NF-κB activation, but also decreases virus replication through an unknown mechanism. miR-302c prevents the translocation of NF-κB from the cytosol to the nucleus by decreasing NF-κB-inducing kinase (NIK) expression [74]. IAV-triggered downregulation of miR-302c leads to increase of nuclear NF-κB and IFN-β expression, and the subsequent inhibition of viral replication. Another group of NF-κB-associated microRNAs is the let-7 family. let-7 is reported to down-regulate the activation of NF-κB by targeting cytokine-inducible Src homology 2-containing protein (CIS) in *Cryptosporidium parvum*-infected cholangiocytes, while let-7f is shown to increase the activity of NF-κB by targeting A20 in *Mycobacterium tuberculosis*-infected macrophages [49,93,94,95]. Virus-induced decrease of let-7 family members was seen in mice with IAV pneumonia and cells infected with r1918, but their effects on the NF-κB pathway remain to be further determined [85,96].

In IAV-infected A549 cells and influenza patients, miR-29 (a, b, and c) are robustly upregulated and involved in the pathway regulating IFN-λ1 expression, leading to increase of IFN-λ1 expression [67,68]. Several key components of the type I IFN signaling pathway, such as interferon-α receptor 1 (IFNAR1) and signal transducer and activator of transcription (STAT2), are speculative targets of miR-200a via in silico analysis. The downregulation of miR-200a and the inverse upregulation of these genes were demonstrated in r1918-infected lungs. Most recently, the PRR-mediated down-regulation of miR-650 was interestingly revealed to modulate the expression of several ISGs, including interferon regulatory factor 3 (IRF3), IFIT2 and MxA. In IAV-infected monocyte-derived dendritic cells (MDDCs), miR-650 directly targets the antiviral ISG MxA. The virus-induced reduction of miR-650 leads to the increase of the MxA and the establishment of an antiviral state in host [61].

miRNAs also participate in modulating the development of immune cells, inflammation and apoptosis. For example, miR-233 is predicted to suppress the key genes in the upstream pathways of cyclic AMP (cAMP)-responsive element binding protein (CREB), an important transcription factor for T-cell development and survival [85,97]. Although the greatly increased expression of miR-233 and the extreme downregulation of the CREB are concomitant in r1918-infected lungs [98,99], more direct evidence is required to verify the regulation and the underlying mechanism. The specific induction of miR-141 by H5N1 causes the decrease of miR-141 target, the cytokine TGF-β2, which acts as an immunosuppressor and a potent pro-inflammatory cytokine. It was thought that the low expression of TGF-β2 in H5N1-infected cells might contribute to severe inflammation [72].The host also uses miRNAs to regulate apoptosis to defend against IAV infection. For example, miR-29c is significantly upregulated in A549 cells after IAV infection, resulting in translational inhibition of its target BCL2L2, an anti-apoptotic member of Bcl-2 family. Therefore, miR-29c promotes the influenza virus-induced apoptosis in A549 cells to suppress viral replication [67]. miR-4276 targets COX6C, a critical part of the intrinsic apoptotic pathway leading to caspase-9 activation. A previous study exhibited that IAV-infected host cells upregulate COX6C expression through significant downregulation of miR-4276 and trigger the cell death of infected cells to diminish virus [60].

During the interaction between host and virus, some host miRNAs are hijacked by the virus to facilitate viral gene expression or progeny production (Figure 1). One strategy taken by IAV is to downregulate the antiviral signaling pathways, transcription factor activity or cytokine expression. For example, IAV utilizes host regulator miR-29c to indirectly down-regulate the NF-κB activity via a mechanism different from the canonical post-transcriptional way. Acting as a decoy, miR-29 directly interacts with the RNA binding protein HuR (human antigen R), and prevents HuR from binding to the 3′-UTR of A20, also known as tumor necrosis factor-α-induced protein 3 (TNFAIP3, the negative regulator of NF-κB activity) and recruiting the RNA degradation complex, RNA-induced silencing complex (RISC) [100]. In IAV-infected A549 cells, virus-induced up-regulation of miR-29c enhances the abundance of A20 transcripts, subsequently decreases the NF-κB activity, and abrogates the expression of downstream antiviral and proinflammatory cytokines, including TNF-α, IFN-β, IL-1β, IL-6, and IL-8 [69]. In IAV-infected primary murine dendritic cells, strongly-induced miR-451 deregulates its target gene YWHAZ/14-3-3ζ. Then, reduced YWHAZ levels result in negative regulation of type I IFN and pro-inflammatory cytokines, such as IL-6, C-C motif chemokine ligand 5 (CCL5) and TNF [62]. Given these cytokines are important proteins in antiviral response, it is reasonable to hypothesize that the virus-induced expression of miR-451 in dendritic cells would be beneficial for the virus. Another strategy taken by virus is to protect its RNAs. In recent research, miR-9 was demonstrated to target MCPIP1, a PilT N-terminus (PIN)-like RNase capable of degrading viral RNA and inhibiting viral genes expression. IAV manipulates this mechanism by up-regulating miR-9 to impair the expression of MCPIP1 and thereby benefit the IAV replication [65]. Additionally, it was observed that during the early stage of IAV infection, the virus induces the downregulation of miR-548an and subsequently increases the abundance of NS1ABP, a host protein that mediates the inhibition of apoptosis and finally facilitates the virus replication [60,63].

### 3.5. miRNAs Inhibit IAV by Directly Suppressing Viral Gene Expression

Several host miRNAs have been found to inhibit IAV replication through directly targeting IAV mRNAs (Figure 1 and Table 1). A previous report discovered that three miRNAs: miR-323, miR-491, and miR-654, were able to decrease H1N1 production in madin darby canine kidney (MDCK) cells via binding to a same conservative site on PB1 mRNA and subsequently inducing mRNA degradation [26]. H1N1-upregulated miR-let-7c is another interesting example, which suppresses virus titers via specifically interacting with virus M1 (+) cRNA/mRNA at the 3′-UTR [77]. The dual functional miR-485 can inhibit the replication of H5N1 virus by binding to the viral RNA polymerase gene PB1, which is required for viral replication in a sequence-specific manner [75]. It is speculated that when increased amounts of the H5N1 virus present in cells, the miR-485 would change its shift from suppressor of RIG-I signaling pathway to the inhibitory role to decrease H5N1 production. Recently, miR-3145 was discovered to inhibit the PB1 expression of three IAV subtypes (pH1N1, H5N1 and H3N2) through in silico analysis and experiments. However, miR-3145 is not constitutively expressed in uninfected human lung A549 cells nor induced by infection [76]. Better understanding of miR-3145 function is required in the future.

Furthermore, the in silico analysis performed by Khongnomnan et al. predicted another 75 miRNAs that might target viral genes of IAV, including miR-216b, miR-3682, miR-4513, miR-4753 and miR-5693 [76]. He et al. also proposed through MiRanda prediction that some host miRNAs (36 from pig and 22 from human) putatively target to the evolutionarily conserved regions of viral NP, NA, HA or *PB2* gene of IAV strains, including swine influenza virus (SIV) and swine-origin 2009 A/H1N1 influenza virus (S-OIV) [101]. These findings provide plentiful clues of miRNAs that might have the potential to be used as novel therapeutics for influenza.

In addition to direct suppression of viral gene expression, another novel mechanism of miRNAs to inhibit virus replication is through regulating the viral protein maturation or activity. Human miR-24, as the representative of this type, is robustly downregulated by the highly pathogenic avian-origin H5N1 strain in A549 cells. The virus-induced decrease of miR-24 benefits the expression of furin, the target of miR-24, and promotes the furin-mediated proteolytic activation of HA precursor [66].

### 3.6. Diagnostic and Therapeutic Applications of miRNAs

It has been revealed that some miRNAs in the serum of influenza patients, including miR-150, miR-29c, miR-145 and miR-22, are associated with severity of the disease caused by pandemic H1N1/2009 [102]. It is worth noting that more experiments have been performed to establish the list of miRNAs detectable in the blood of influenza patients [55,57,89]. It was observed that in whole blood of H1N1 patients, miR-1260, miR-26a, miR-335, miR-576-3p, miR-628-3p and miR-664 are differentially expressed [57]. For pandemic H1N1/2009 virus-infected patients, miRNAs with significant potential diagnostic value are detected not only in the peripheral blood mononuclear cells (PBMCs) (miR-31, miR-29a and miR-148a) [55], but also in the serum (miR-150, miR-29c, miR-145 and miR-22) [102]. In serum of H7N9 patients, levels of miR-17, miR-20a, miR-106a and miR-376c were significantly elevated, as compared with healthy individuals [89]. These data suggest that particular miRNAs could be used as biomarkers of influenza.

Moreover, an in vivo study displayed that in IAV-challenged pigs, the number and type of differentially expressed circulating miRNAs in blood leukocytes are dynamically changed with the infection time [103]. Importantly, by using the specific groups of miRNAs, a simple assay was successfully established for the diagnosis of influenza A and B [104]. Interestingly, an attenuated IAV strain has been generated through a genetic insertion of a specific miRNA recognition sequence to the IAV PB1 genomic segment. Such an IAV strain may be utilized as a safe and effective influenza virus vaccine [105,106]. In addition, a plant honeysuckle-encoded miR-2911 was reported to target various IAVs, including H1N1, H5N1 and H7N9. Its function to significantly inhibit viral replication was manifested through in vitro and in vivo experiments. Extracted or synthetic miR-2911 was found to dramatically reduce H1N1-encoded PB2 and NS1 protein expression [78]. Taken together, these data suggest that the applications of these particular miRNAs might be helpful for the surveillance on highly pathogenic IAV outbreak or other pandemic IAV infection, and for diagnostic and therapeutic tools. However, more adequate and profound investigations are required in the future [78].

## 4. Involvement of lncRNAs in Anti-IAV Innate Immunity

Increasing studies have provided evidence that lncRNAs are important regulators involved in antiviral response. The abundances of lncRNAs are significantly changed during viral infection with both DNA virus (e.g., herpesvirus and adenovirus) and RNA virus (e.g., HCV, HIV, and IAV). However, the functions and underlying mechanisms of these differentially expressed lncRNAs remain largely unknown. From the limited literature, lncRNAs that are proven to be involved in the virus-host interaction can be classified to several groups: most of them are associated with the production of IFNs, the IFNs-stimulated Janus kinase-signal transducer and activator of transcription (JAK-STAT) signaling pathway, and the expression of ISGs; some are related to the activation or translocation of transcription factors; some regulate other antiviral proteins or pathways [13,35]. The IAV-induced differential expression of hundreds of lncRNAs has been reviewed in details by else [13,16,107,108]. Here we concentrate on the roles played by functional lncRNAs in the IAV pathogenesis.

Negative regulator of anti-viral (NRAV) was described as a constitutively expressed lncRNA which is downregulated in a virus-dose dependent manner during the infection with IAV, Sendai virus (SeV) or herpes simplex virus (HSV) [79]. The decrease of NRAV leads to up-regulation of the MxA and IFITM3 expression. Downregulation of NRAV does not affect the JAK-STAT signaling pathway, but markedly increase an active mark of transcription (histone 3 lysine 4 trimethylation, H3K4me3) and significantly declines a repression signal of transcription (histone 3 lysine 27 trimethylation, H3K27me3) at the transcription level of MxA and IFITM3 genes. This histone modification in the gene transcription site is the cornerstone of the alteration in the MxA and IFITM3 expression. This was confirmed in NRAV transgenic overexpressing mice when compared to control wild types mice infected with IAV. The transgenic mice showed much higher sensitivity to IAV infection with higher virus titers in lung, weight loss and mortalities. In NRAV transgenic mice, several critical ISGs were downregulated, including IFIT2, IFIT3, 2′-5′-oligoadenylate synthetase-like protein (OASL), Mx1 and IFITM3. NRAV can negatively regulate the initial transcription of these genes by affecting their histone modifications.

NEAT1 is upregulated by stress, IAV and HSV infection, or poly I:C treatment. As an essential component for the formation of paraspeckles, it is involved in the expression of various genes related to innate immunity [80,81,109,110]. The subnuclear paraspeckle structures are formed dynamically in response to stress or cellular metabolic changes, depending on transcription and RNA polymerase II [111]. Wang et al. reported recently that the expression of NEAT1 induced by HSV is dependent on STAT3. In turn, NEAT1 co-locates with phosphorylated STAT3 in paraspeckles and promotes the expression of IL-8 [80], RNA-specific adenosine deaminase B2 (ADARB2) [112] and HSV viral genes [81] by binding with SFPQ/PSF and other paraspeckle protein components, 54 kDa nuclear RNA-binding protein (P54nrb) and paraspeckle protein 1 (PSPC1). When NEAT1 is in a low level, SFPQ/PSF acts as a repressor for the promoter of IL-8 and HSV viral genes. During IAV and HSV infection, the expression of the NEAT1 is greatly induced, leading to the relocation of the SFPQ/PSF to the paraspeckles and resulting in the activation of the antiviral gene IL-8. Interestingly, SFPQ/PSF was also shown to be important for IAV mRNA polyadenylation [113]. Therefore, NEAT1 is a crucial innate immune molecule with dual functions that on the one hand is a critical part of antiviral response to increase the expression of immune cytokine, but on the other hand can be hijacked by viruses to facilitate viral gene expression and viral replication.

BISPR is one of the IFNα2-stimulated lncRNAs. It was shown to be greatly induced by the infection of IAV NS1 mutant strain [107,114]. It regulates IFN secretion in a location-specific manner through upregulating BST2 expression in response to the viral infection [114]. Inhibition of the BISPR expression using siRNA decreases the levels of ISG BST2 without affecting other the expression of other genes, while overexpression of the BISPR lncRNA was shown to up-regulate the BST2 [107,114,115]. However, the underlying mechanism of BISPR to stimulate the adjacent BST2 transcription still needed to be defined.

VIN, termed virus inducible lincRNA, is upregulated by IAV infection such as H1N1, H3N2 and H7N7 strains, as well as VSV, but is not induced by influenza B virus infection, treatment with RNA mimics, or IFN-β [82]. The abolition of nucleus-located VIN resulted in significant decrease of the virus replication and viral gene expression (HA, NP, NS1 and M2), indicating that VIN is utilized by virus to guarantee its successful propagation [82]. How VIN promotes viral gene expression and replication remains to be clarified. 

Eosinophil granule ontogeny transcript (EGOT) is a recently identified lncRNA acting as a negative regulator of the antiviral response [108]. The induction of EGOT by hepatitis C virus (HCV) infection is through the RIG-I and PKR-dependent signaling pathway. After sensing viral RNAs, RIG-I and PKR activate NF-κB which initiates the expression of EGOT. EGOT also can be induced by other viruses including IAV and Semliki Forest virus (SFV). Disruption of EGOT in host cell leads to higher expression of several ISGs and decreases viral replication. Therefore, the HCV-mediated up-regulation of EGOT is a strategy of the virus to suppress the IFN-signaling pathway and thus promote viral production.

In addition, many lncRNAs are recognized as involved in antiviral response to infection with viruses other than influenza virus. For instance, lncRNA–CMPK2, an IFN-stimulated lncRNA, has been identified as a negative regulator of the anti-HCV innate response [116]. However, although there is increasing number of lncRNAs that are identified as novel regulators of host–virus interaction, the precise mechanisms underlying their functioning during viral pathogenesis are still unclear.

## 5. vtRNAs Are Involved in IAV Pathogenesis by Inhibiting PKR Activation

Strikingly, previous studies showed that the four members of human vtRNAs, vtRNA1-1, 1-2, 1-3, and vtRNA2-1,were robustly upregulated in IAV infected human cells, and similar results were obtained from IAV-challenged mouse lungs [31,35]. Importantly, inhibition of the expression of vtRNAs causes impaired IAV infection, while forced-expression of vtRNAs greatly increases viral propagation [35]. Further, vtRNAs are found to promote viral replication through repressing the activation of protein kinase R (PKR), a vital component of host innate immunity against viral infection, and subsequently inhibit the antiviral interferon response [34,35,117]. Interestingly, it was also revealed that vtRNAs were required for the IAV protein NS1-induced inhibition of PKR activity. Given vtRNAs are also induced by other viruses (SeV and HSV-1), these results provide a novel mechanism by which viruses escape the PKR-mediated antiviral response through up-regulation of host vtRNAs.

## 6. Conclusions

It is well known that ncRNAs play important roles in a wide range of cellular processes. Therefore, dysregulation of ncRNAs is closely associated with many diseases, including cancer and auto-immune diseases. Influenza caused by IAV infection is one of the most serious threats to human public health. However, many aspects of the interaction between IAV and host have not been fully characterized. Over the past ten years, hundreds of ncRNAs have been detected to be differentially expressed during the IAV infection. Importantly, many of them have been identified to be involved in the host immune response to the virus infection through regulating almost every critical step of antiviral immunity, including PRR-dependent signaling pathways for sensing the virus, activation of transcription factors such as NF-κB, production of various IFNs and cytokines, induction of ISG expression, host cell development and apoptosis. Interestingly, some of them modulate the viral gene expression directly. On the one hand, host ncRNAs are implicated in defending against viral invasion. On the other hand, some ncRNAs are hijacked by the virus for use for infection and replication. Taken together, these findings have substantially indicated that ncRNAs act as key regulators of host–IAV interaction and are critically involved in IAV pathogenesis. Better understanding of the differential expression of these ncRNAs, their functioning and the precise mechanisms underlying their regulation during the IAV infection would promote the utilization of them as novel targets for therapeutics or as biomarkers of the disease.

## Figures and Tables

**Table 1:** Functional involvement of ncRNAs in regulation of the influenza virus infection.

ncRNAs	Stimuli	Differential Expression	Functions/Mechanisms	References
microRNA (miR)-7, miR-132, miR-187, miR-200c, and miR-1275	H1N1	Up	cause down-regulation of antiviral proteins such as IL-1R-associated kinase 1 (IRAK1)and mitogen-activated protein kinase 3 (MAPK3)	[64]
miR-9	H1N1 and H3N2	Up	promotes IAV replication through suppression of monocyte chemoattractant protein 1-induced protein 1 (MCPIP1)	[65]
miR-24	H5N1	Down	governs furin-mediated proteolytic activation of hemagglutinin precursor (HA0) glycoproteins and production of infectious virions	[66]
miR-29	H3N2	Up	suppresses DNA methyltransferase (DNMT)3a/3b activity and induces expression of cyclooxygenase-2 (COX2) and IFN-λ1	[67,68]
miR-29c	H3N2 and H1N1	Up	induces virus-mediated apoptosis through repression of antiapoptotic factors B-cell lymphoma 2 like 2 (BCL2L2), and inhibits the innate immune response through protection of deubiquitinating enzyme A20 mRNA	[67,69]
miR-33a	H1N1, H9N2 and H3N2	Up	disturbs IAV replication by targeting archain 1 (ARCN1) and inhibiting viral ribonucleoprotein activity	[70]
miR-136	H5N1	Up	acts as an immune agonist of RIG-I, causing interleukin-6 (IL-6) and IFN-β accumulation	[71]
miR-141	H5N1	Up	suppresses the expression of transforming growth factor β2 (TGF-β2) mRNA	[72]
miR-146a	H1N1and H3N2	Up	suppressesIRAK1, MAPK3, tumor necrosis factor (TNF) receptor-associated factor 6 (TRAF6) expression and nuclear factor kappa-light-chain-enhancer of activated B cells (NF-κB) activation, and decreases virus replication	[64,73]
miR-302c	H3N2	Down	prevents the translocation of NF-κB from the cytosol to the nucleus, leading to the suppression of IFN-β expression	[74]
miR-323, miR-491, miR-654	H1N1	NA	bind with the polymerase basic 1 (*PB1*) gene, and downregulates PB1 expression through mRNA degradation	[26]
miR-451	H1N1	Up	negatively regulates the levels of YWHAZ protein which controls the activity of two negative regulators forkhead box O3 (FOXO3) and zinc finger protein 36 (ZFP36) of cytokine production	[62]
miR-485	H5N1	Up	exhibits bispecificity, targeting RIG-I with a low abundance of H5N1 virus and targeting PB1 with increased amounts of the H5N1 virus	[75]
miR-548an	H1N1	Down	triggers the overexpression of an anti-apoptotic protein non-structural-1A binding protein (NS1ABP)	[63]
miR-650	H1N1	Down	directly targets the antiviral ISG myxovirus resistance protein 1 (MxA) and fine-tunes its expression	[61]
miR-3145	pH1N1, H5N1 and H3N2	NA	inhibits IAV replication by targeting and silencing viral *PB1* gene	[76]
miR-4276	H1N1, H3N2	Down	downregulates the expression of apoptotic protein cytochrome c oxidase VIc (COX6C)	[60]
let-7c	H1N1	Up	inhibits matrix protein 1 (M1) expression	[77]
plant miR-2911	NA	NA	suppresses H1N1,H5N1 and H7N9 replication, and inhibits H1N1-encoded PB2 and non-structural protein 1 (NS1) expression	[78]
NRAV	H1N1	Down	negatively modulates antiviral responses through suppressing of ISGs‘ transcription, including interferon-induced protein with tetratricopeptide repeats 2 (IFIT2), IFIT3, 2'-5'-oligoadenylate synthetase-like protein (OASL), MxA and interferon-induced transmembrane protein 3 (IFITM3)	[79]
NEAT1	H1NI	Up	regulates the expression of IL-8 through sequestring splicing factor proline-glutamine rich (SFPQ/PSF) in paraspeckles	[80,81]
virus inducible lincRNA (VIN)	H1N1, H3N2 and H7N7	Up	is induced by IAV to benefit viral replication and viral gene expression	[82,83]
vtRNAs	H1N1	Up	promote viral replication through repressing the activation of PKR and the subsequent antiviral interferon response	[35]

Up, upregulation; Down, downregulation; NA, not available.

## Abbreviations

ncRNA	non-coding RNA
lncRNA	long non-coding RNA
IAV	influenza A virus
PAMP	pathogen-associated molecular pattern
PRR	pattern recognition receptor
TLR	toll-like receptor
RIG-I	retinoic acid-inducible gene 1
MDA5	melanoma differentiation-associated gene 5
IFN	interferon
ISG	IFN-stimulated gene
vtRNA	vault RNA
JAK-STAT	Janus kinase-signal transducer and activator of transcription
TNF	tumor necrosis factor
NF-κB	nuclear factor κ-light-chain-enhancer of activated B cells
IRF	interferon regulatory factor
IFIT	interferon-induced protein with tetratricopeptide repeats
OASL	2′-5′-oligoadenylate synthetase-like protein
MxA	Myxovirus resistance protein 1
IFITM	interferon-induced transmembrane protein
TGF	transforming growth factor
NRAV	negative regulator of anti-viral
NEAT1	nuclear enriched abundant transcript 1
VIN	virus inducible lincRNA
SFPQ/PSF	splicing factor proline/glutamine-rich
